# Conspiracy Theories, Psychological Distress, and Sympathy for Violent Radicalization in Young Adults during the COVID-19 Pandemic: A Cross-Sectional Study

**DOI:** 10.3390/ijerph18157846

**Published:** 2021-07-24

**Authors:** Anna Levinsson, Diana Miconi, Zhiyin Li, Rochelle L. Frounfelker, Cécile Rousseau

**Affiliations:** 1 Division of Social and Cultural Psychiatry, McGill University, CLSC Parc-Extension, 7085 Hutchison, Montréal, QC H3N 1Y9, Canada; diana.miconi@mail.mcgill.ca (D.M.); rochelle.frounfelker@mail.mcgill.ca (R.L.F.); cecile.rousseau@mcgill.ca (C.R.); 2 Department of Epidemiology, Biostatistics, and Occupational Health, McGill University, 1020 Pine Ave. W, Montreal, QC H3A 1A2, Canada; zhi.yin.li@mail.mcgill.ca

**Keywords:** COVID-19 conspiracy theories, psychological distress, violent radicalization

## Abstract

The COVID-19 pandemic has spread uncertainty, promoted psychological distress, and fueled interpersonal conflict. The concomitant upsurge in endorsement of COVID-19 conspiracy theories is worrisome because they are associated with both non-adherence to public health guidelines and intention to commit violence. This study investigates associations between endorsement of COVID-19 conspiracy theories, support for violent radicalization (VR) and psychological distress among young adults in Canada. We hypothesized that (a) endorsement of COVID-19 conspiracy theories is positively associated with support for VR, and (b) psychological distress modifies the relationship between COVID-19 conspiracy theories and support for VR. A total of 6003 participants aged 18–35 years old residing in four major Canadian cities completed an online survey between 16 October 2020 and 17 November 2020, that included questions about endorsement of COVID-19 conspiracy theories, support for VR, psychological distress, and socio-economic status. Endorsement of conspiracy theories was associated with support for VR in multivariate regression (β = 0.88, 95% confidence interval (CI) 0.80–0.96). There is a significant interaction effect between endorsement of COVID-19 conspiracy theories and psychological distress (β = 0.49, 95% CI 0.40–0.57). The magnitude of the association was stronger in individuals reporting high psychological distress (β = 1.36, 95% CI 1.26–1.46) compared to those reporting low psychological distress (β = 0.47, 95% CI 0.35–0.59). The association between endorsement of COVID-19 conspiracy theories and VR represents a public health challenge requiring immediate attention. The interaction with psychological distress suggests that policy efforts should combine communication and psychological strategies to mitigate the legitimation of violence.

## 1. Introduction

Countering violent radicalization (VR), defined as an “individual or collective process whereby normal practices of dialogue, compromise and tolerance between groups/individuals with diverging interests are abandoned and one or more groups/individuals engage in violent actions to reach a specific goal,” [1] is a public health priority. Ideologically motivated violence is a matter of public health because acts of terror, hate incidents, and hate crimes have serious physical and mental health consequences not only for those most proximally affected by violence, but also impacting the wellbeing of the broader population [2]. In the past, efforts to counter VR were primarily concerned with violent actions of lone actors and marginalized extremist groups; now, radical and violent ideologies have become mainstream and infiltrated the highest levels of government in countries around the world [3].

This past year, the COVID-19 pandemic has impacted and interacted with VR in troubling ways. The pandemic has accentuated structural violence, including systemic racism and socio-economic disparities [4], which exacerbate social polarization and support for VR [5,6]. Extremist groups have, and continue to, capitalize on the pandemic to spread disinformation that scapegoat marginalized communities and endorse acts of violence [7]. Political leaders around the world, including in the United States, exploit COVID-19 related fear and anger in the populace to consolidate power and promote anti-democratic agendas [3]. 

One strategy used by extremists to sow dissent is to endorse conspiracy theories about the origins of the virus and authorities’ handling of the pandemic [8,9]. COVID-19 conspiracy theory beliefs include ideas such as: (1) contrary to information provided by governments, the virus is of unnatural origin; (2) the spread of the virus is encouraged by pharmaceutical companies for financial gain, and; (3) the virus is a vehicle of the Chinese government [10]. Endorsement of conspiracy theories such as these fuel distrust in government and public health authorities and are associated with intentions to follow public health guidelines (e.g., anti-vaccine beliefs) [10,11]. Furthermore, COVID-19 conspiracist ideation is positively associated with the justification, willingness and intent to be violent, findings that are aligned with previous research linking general endorsement of conspiracy theories and violent action [12]. 

COVID-19 conspiracist ideation is associated with greater anxiety and feelings of powerlessness [13], both indicators of psychological distress, and the impact of the pandemic on psychological distress is becoming well established [14]. Anxious and depressive symptoms are associated with fear of the virus and with the collateral damages stemming from the confinement, and in particular from income loss and social isolation. The pandemic has exacerbated pre-existing social inequalities and mounting evidence indicates that COVID-related psychological distress disproportionately affects minority groups and vulnerable communities [15]. Conspiracy beliefs may provide a mechanism to empower individuals by allowing them to adopt narratives that explain and reduce the current uncertainties and distress associated with powerlessness. As such, conspiracy beliefs about the pandemic may provide individuals with an opportunity to feel both more in control in times of high uncertainty and more connected to a community of individuals who share the same emotional experience and beliefs [12,16].

Throughout history, conspiracy theories have been a part of pandemics and epidemics. As far back as the medieval black plague, conspiracy theories proliferated to provide meaning and assign responsibility of the perceived threat to outgroup individuals, either marginal or strangers [17,18]. Thus, narratives designating an out-group enemy were widespread before the COVID-19 health emergency, and are now associated with an exacerbation of social inequalities and an absence of future perspectives [19]. In the past year, high levels of uncertainty and distress brought about by the pandemic have fueled an upsurge in social polarization.

**Figure 1:**
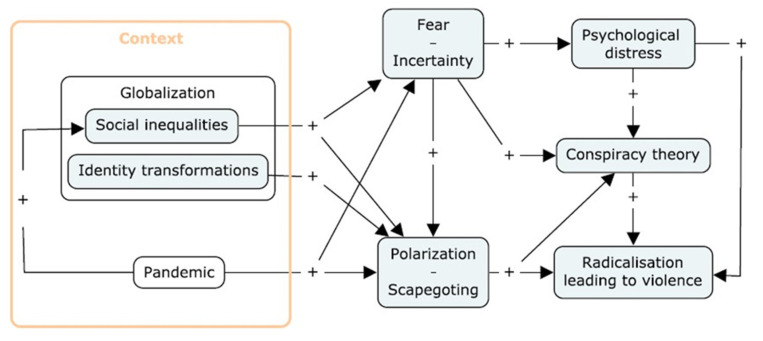
Radicalization leading to violence, endorsement of COVID-19 conspiracy theories and psychological distress in relational context.

The association between psychological distress and support for VR has been repeatedly corroborated [20,21]; in contrast, to date no research has explored the potential moderation of the association between COVID-19 conspiracist ideation and support for VR by psychological distress. Studying the relationships between conspiracy theories, attitudes legitimizing VR, and psychological distress is crucial in developing public health communication and prevention programs which take into account the mental health component of these social dynamics. Results from such studies could contribute to the identification of tools to mitigate the conflicts and violence associated with the present context. We propose a theoretical model (Figure 1) which contextualizes the relationship between support for VR, endorsement of COVID-19 conspiracy theories and psychological distress.

This study aims to investigate associations between endorsement of COVID-19 conspiracy theories, psychological distress and sympathy for VR in a large sample of young adults living in four Canadian urban settings (i.e., Calgary, Edmonton, Montreal and Toronto). We hypothesised that (a) support for VR is positively associated with endorsement of COVID-19 conspiracy theories, and (b) there is an interaction effect of psychological distress and endorsement of COVID-19 conspiracy theories on support for VR.

## 2. Materials and Methods

### 2.1. Description of the Sample

In total, 6003 individuals aged 18–35 completed an on-line survey, of which 54.8% were women (see Table 1 for a detailed description of the sample).

The online survey targeted young adults in large cities in Quebec, Ontario and Alberta. We anticipated a sample size of 2000 participants in each province for a total of 6000 participants. Data collection took place between 16 October 2020 and 17 November 2020. The total response rate for the survey was 19% and 19%, 18%, 19% and 22% in Calgary, Edmonton, Montreal and Toronto, respectively. Inclusion criteria for participants were: aged between 18 and 35 years, and residents of Montreal, Toronto, Calgary or Edmonton. Exclusion criteria were: cognitive deficit or other disability that would prevent an individual from providing informed consent, and not speaking English or French (the languages in which the survey was administered). Participants are all registered in the AskingCanadians pool, a Delvinia Technology Inc. online data collection firm with access to more than one million Canadian professionals and consumers who are nationally representative by region and monitored against Statistics Canada. The firm emailed potential participants an introductory message with a hyperlink to the survey. Participants received a gift card valued at 2.50$ according to how much time they dedicated to the survey. Ethics approval was obtained by X (BLINDED FOR REVIEW) before initiating the study, and all participants provided an electronic informed consent.

### 2.2. Measures

#### 2.2.1. Endorsement of Conspiracy Theories

Endorsement of COVID-19-related conspiracy theories was assessed with questions asking participants to rate, on a Likert scale from 1 = do not agree to 5 = agree completely, their level of agreement with four statements adapted from Freeman et al. [22]: “The government is misleading the public about the cause of the Coronavirus”, “The spread of the Coronavirus is a deliberate attempt by a group of powerful people to gain control”, “Coronavirus is a bioweapon developed by China to destroy the West”, and “The mainstream media is deliberately feeding us misinformation about the Coronavirus and lockdown”. The Cronbach alpha for the total score was 0.88. 

#### 2.2.2. Attitudes toward Violent Radicalization

The Sympathies for Radicalization scale (SyfoR) [23] consists of questions related to nine acts of protest ranging from nonviolent (e.g., take part in non-violent political protests) to progressively more extreme acts (e.g., use of suicide bombs to fight against injustices). Subjects are asked to rate their attitude towards these acts on a 7-point Likert scale (1 = completely condemn to 7 = completely sympathize) with a higher score meaning greater support for VR. A total score (range 8–56) of sympathy for radicalization was used in this study (excluding the non-violent protest item). The SyfoR has been adapted to Canadian contexts [24]. Cronbach’s alpha in this study was 0.97.

The Radicalism Intention Scale (RIS) is a subscale of the validated Activism and Radicalism Intention Scales (ARIS). A previous validation with ethnically diverse populations yielded adequate internal consistency and discriminant validity [25]. The RIS assesses an individual’s willingness to support illegal and violent behaviour in the name of one’s in-group or organisation. It is composed of four items rated on a 7-point Likert scale (1 = completely disagree to 7 = completely agree) with a higher total score indicating more support for VR. The total score (range 4–28) was used for sensitivity analyses in this study and Cronbach’s alpha was 0.95.

#### 2.2.3. Psychological Distress

The Hopkins Symptom Checklist-25 (HSCL-25) is a self-report questionnaire aimed at screening for levels of anxiety and depression. Items are rated on a Likert scale from 1 (not at all) to 4 (extremely), and a total score is obtained by computing the mean of all items. The clinical cut-off is set at 1.75 (score range from 1 to 4). This means that an individual with a score of 1.75 or more can be considered as having high psychological distress. The HSCL-25′s psychometric qualities and transcultural validity have been well established among different cultural groups [26,27]. Cronbach’s alpha in this study was 0.98.

#### 2.2.4. Sociodemographic Variables

Participants self-reported age, city of residence (Montreal, Calgary, Edmonton, Toronto), gender (woman, man or gender-diverse), and immigrant generation (first-, second- and third and above-generation immigrant). The financial problems variable was collected using the question “Presently in your household, are you experiencing difficulties related to lack of money?” (Not at all, some, a moderate amount, a lot), and educational level was collected with the question “What is the highest grade you completed?” (high school or less, technical degree or some college/university, university degree and above).

### 2.3. Statistical Analysis

Missing data were imputed with multiple imputation chained equations, R-package *mice*, using 5 imputed datasets [28]. For Table 2, *p*-values for differences in mean scores between categories were calculated using ANOVA. Effect size is reported as η2. Multivariate regressions and moderation analysis were adjusted for self-reported gender, age, city of residence, reported level of financial problems, educational level and immigrant generation. First, the association between VR and endorsement of COVID-19 conspiracy theories was established using linear regression adjusted for the listed covariates. Second, moderation was identified using an interaction term for endorsement of COVID-19 conspiracy theories and the continuous mean score for psychological distress in the regression of sympathy for VR. Finally, the association between endorsement of COVID-19 conspiracy theories and sympathy for VR was estimated in strata below and above the clinical cut-off for HSCL-25.

## 3. Results

At the descriptive level, mean total SyfoR and endorsement of COVID-19 conspiracy theories scores were higher for self-reported men compared with women, and lower in first- and second-generation immigrants compared with individuals from families residing in Canada for three or more generations. A majority of respondents (69.1%) reported no or little financial problems, but those who did also had higher levels of sympathy for VR, endorsement of COVID-19 conspiracy theories, and psychological distress. Regarding educational level, sympathy for VR, endorsement of COVID-19 conspiracy theories, and psychological distress were highest for individuals with a technical degree or some college/university. Scores on sympathy for VR, endorsement of COVID-19 conspiracy theories, and psychological distress were all higher in younger individuals (age 18–25 compared to 26–35). Among the four cities, participants in Montreal reported the highest unadjusted scores on the SyfoR, endorsement of conspiracy theories and psychological distress. Almost half of the total sample reported psychological distress mean scores above the clinical cut-off 1.75. In Montreal, this proportion was 56.1%.

In multivariate regression, endorsement of conspiracy theories was significantly associated with SyfoR scores (β = 0.88, 95% confidence interval (CI) 0.80–0.96). (Table 3) First generation immigrants had a significantly lower SyfoR score than third or more generation, while there was no significant difference for second generation immigrants. Higher education was significantly associated with higher SyfoR scores. Different to the descriptive analyses, SyfoR scores were highest for individuals with university-level education. However, moderate financial problems were significantly associated with higher SyfoR scores, indicating a non-linear association between socio-economic status and support for VR in the current context. 

### Moderation Analyses

**Figure 2:**
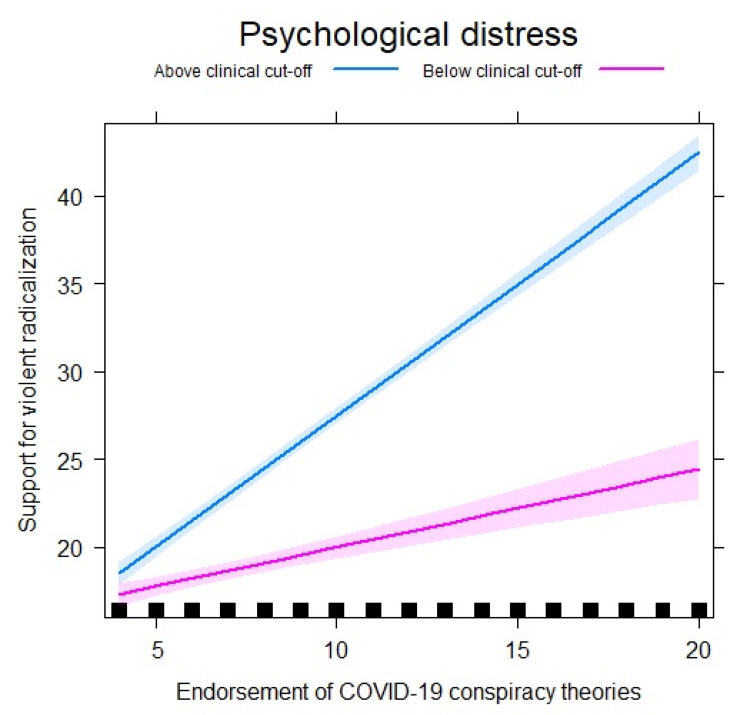
Psychological distress as moderator of the association between sympathy for violent radicalization and endorsement of COVID-19 conspiracy theories.

A significant interaction (moderation) was seen between endorsement of COVID-19 conspiracy theories and psychological distress continuous mean scores (β = 0.49, 95% CI 0.40–0.47). (Table 4) The magnitude of the association between endorsement of COVID-19 conspiracy theories and SyfoR was greater in individuals reporting high psychological distress (β = 1.36, 95% CI 1.26–1.46) compared to those reporting low psychological distress (β = 0.47, 95% CI 0.35–0.59). The moderation effect of psychological distress on the association between sympathy for VR and endorsement of COVID-19 conspiracy theories is also illustrated in Figure 2.

Sensitivity analyses using the RIS as outcome showed results in the same direction as for SyfoR (Supplemental Tables S1 and S2).

## 4. Discussion

To the best of our knowledge, this is the first study to provide evidence of a worrisome phenomenon: the association between endorsement of COVID-19 conspiracy beliefs and support for VR during the present health emergency among a large sample of young adults in four different urban settings in Canada. Further, the study shows moderation of this association by psychological distress. 

Prior research suggests that endorsement of COVID-19 conspiracy theories is problematic from a public health perspective, because it may hinder following public health guidelines during the pandemic [8, 9, 10]. Our results highlight another concerning public health issue related to the endorsement of conspiracy theories, that of increased support for VR [2]. During the pandemic, research has reported instances of support for, and engagement in, VR fueled by COVID-19 conspiracy theories. Most notably, the association of the virus with China has resulted in an increase in hate crimes and violence against individuals who identify as Asian [29]. There are also concerns that a believed association between COVID-19 and 5G technology motivated arson attacks against telecommunication infrastructure [12]. Nonetheless, to our knowledge, to date no multi-site empirical study has explored and demonstrated a relationship between psychological distress, COVID-19 conspiracy theories and legitimation of violence. The issue is urgent, as the global spread of COVID-19 conspiracy theories indicates a risk of a concomitant increase in levels of both sympathy for and acts of VR. An upsurge in levels of sympathy for VR may result in an increase in discrimination, hate crimes and incidents, as well as more deadly attacks from either lone actors or organized groups, particularly militias, encouraged by this shift in population attitudes. Our finding that the association of conspiracy theories and support for VR was moderated by psychological distress supports the hypothesis that symptoms of anxiety and depression interact with conspiracy beliefs, which are in turn associated with higher support for VR, highlighting the crucial role of supporting mental health among young people during the pandemic.

Aligned with previous North American studies of VR, support for VR scores were lower among women, older participants, and first-generation immigrants. Of importance, the same socio-demographic factors emerged as protective in association with endorsement of COVID-19 conspiracy theories, further highlighting commonalities between the two phenomena. Thus, prevention and intervention programs to reduce both conspiracy beliefs and support for VR should support young adult men and gender minorities, and target majority groups rather than immigrant groups. 

In this study, the non-linear association between socio-economic status and support for VR is noteworthy. Despite the importance of social inequities as a driver of VR, the literature has traditionally reported a weak association between poverty and this outcome [30]. During the pandemic, many have experienced significant financial difficulties, yet our results associate moderate but not severe financial difficulties with VR. This suggests that, while in a situation where survival is at stake, it may sometimes be difficult to engage in resistance or dissent; however, this also adds to the documentation of the pandemic-related upsurge in bitterness associated with the exacerbation of socio-economic inequities.

## 5. Limitations

There are several limitations to this study. First, this is a cross-sectional study; thus we cannot make causal claims and, similarly to previous research, have not been able to ascertain the temporality of psychological distress and conspiracist ideation [13]. Nonetheless, our results are consistent for both SyfoR and RIS, where the association between sympathy for VR and COVID-19 conspiracist ideation is significantly stronger in the stratum with psychological distress mean score above the clinical cut-off. Second, the overall response rate in this study was 19%. As such, there is considerable risk of selection bias that could over- or underestimate the relationship between COVID conspiracy theories and VR. For instance, if people who endorse conspiracy theories and support for VR were less likely to complete the survey, we may be underestimating the relationship between these variables. On-line surveys also present limitations as compared to face-to-face interviews, such as misunderstanding of study questions. In addition, online surveys often show an overrepresentation of individuals with higher education. However, an online survey is advantageous for research specific to conspiracy theories and VR in that it: (1) guarantees anonymity and increases the likelihood that subjects will provide accurate, sensitive information and (2) is attractive to the 18–35 age group and cultural communities who may distrust institutions and authorities [31,32]. Several studies have examined the validity and test–retest reliability of online self-administered survey instruments and found that overall psychometric qualities were satisfactory and comparable to traditional versions. Third, participants in this study were all living in four of the major Canadian urban settings, thus limiting the generalizability of results to young people living in rural areas within North America. Finally, in order to establish whether different types of conspiracy theories are associated with support for VR, more fine-grained measures regarding conspiracy theories are needed. 

## 6. Conclusions

Given our findings, we argue that public health communication strategies regarding COVID-19, which thus far have focused predominantly on cognitive dimensions of disinformation (i.e., deconstructing conspiracy Table 1 theories), should integrate, consider, and target the underlying emotional components associated with the endorsement of conspiracy beliefs. Direct confrontation of conspiracy beliefs is at best ineffective, and at worst harmful [13]. Alternative communication strategies are urgently needed to mitigate social polarization and its violent consequences. They may build on democratic health communication approaches which have been shown efficient during the pandemic [33]. In addition, an increased policy effort to improve feelings of safety and mental wellbeing in young populations during the COVID-19 pandemic is warranted. This may be done through a public health approach targeting social determinants of youth’s wellbeing through their community environments at school or at work. Such efforts should, for instance, aim to mitigate pandemic-related financial uncertainties, decrease the social isolation stemming from social distancing and confinement measures and legitimate the collective discontent associated with the growing inequities revealed by the pandemic. Furthermore, an effort in terms of digital literacy and an increase in visibility and availability of psychological support in the community, i.e., an increased presence of health care services, may be helpful. With the end of the pandemic still not in sight, phenomena of conspiracy theories and VR, which began prior to the COVID-19 virus, should be a priority and be monitored to maintain a proactive prevention agenda rather than a reactive crisis resolution stance, which unfortunately too often prevails in the field of interpersonal violence. 

## Figures and Tables

**Table 1:** Descriptive statistics of participants.

Variable	*n* (%)
Self-reported gender	
Woman	3292 (54.8%)
Man	2646 (44.1%)
Gender-diverse	30 (0.5%)
Missing	35 (0.6%)
Psychological distress	
≤1.75	2441 (40.7%)
>1.75	2974 (49.5%)
Missing	588 (9.8%)
City	
Montreal	2000 (33.3%)
Calgary	1002 (16.7%)
Edmonton	1000 (16.7%)
Toronto	2001 (33.3%)
Financial problems	
Not at all	1963 (32.70%)
A little	2184 (36.4%)
Moderate	896 (14.9%)
A lot	769 (12.8%)
Missing	191 (3.2%)
Education	
High school or less	1267 (21.1%)
Apprenticeship, technical institute, trade or vocational school, college, CEGEP or other non-university certificate or diploma,	1741 (29.0%)
University certificate, diploma or degree	2892 (48.2%)
Missing	103 (1.7%)
Immigration status	
First generation	1454 (24.2%)
Second generation	1577 (26.3%)
Third generation or more	2872 (47.8%)
Missing	100 (1.7%)
	mean (SD) min, max, % missing
Age	26.72 (4.53) 18.00, 35.00, 0.0%
Psychological distress	2.00 (.79) 1.00, 4.00, 9.8%
Endorsement of conspiracy theories	8.78 (4.87) 4.00, 20.00, 7.7%
Sympathy for violent radicalisation (SyfoR)	23.72 (13.86) 8.00, 56.00, 7.9%
Radicalism Intention Scale (RIS)	13.87 (7.40) 4.00, 28.00, 9.3%

**Table 2:** Descriptive statistics of study variables: SyfoR, endorsement of COVID-19 conspiracy theories and psychological distress.

	SyfoR, Total Score			Endorsement of COVID-19 Conspiracy Theories, Total Score			Psychological Distress, Mean Score		
Variable	Mean (SD)	*p*-Value	η2	Mean (SD)	*p*-Value	η2	Mean (SD)	*p*-Value	η2
Self-reported gender		<0.0001	0.04		<0.0001	0.02		0.01	0.002
Woman	21.29 (12.42)			8.24 (4.49)			2.00 (0.71)		
Man	26.62 (14.96)			9.45 (5.23)			1.99 (0.87)		
Gender-diverse	30.42 (10.19)			6.21 (3.31)			2.43 (0.74)		
Age		<0.0001	0.01		<0.0001	0.004		<0.0001	0.02
18–25	25.81 (13.84)			9.17 (4.92)			2.15 (0.82)		
26–35	22.41 (13.72)			8.53 (4.83)			1.91 (0.75)		
City of residence		<0.0001	0.01		<0.0001	0.03		<0.0001	0.03
Calgary	23.66 (12.80)			8.32 (4.57)			1.90 (0.73)		
Edmonton	24.77 (13.62)			8.90 (4.96)			2.05 (0.82)		
Montreal	25.11 (15.91)			9.90 (5.31)			2.16 (0.87)		
Toronto	21.77 (11.86)			7.80 (4.23)			1.86 (0.68)		
Financial problems		<0.0001	0.10		<0.0001	0.13		<0.0001	0.25
Not at all	19.51 (11.14)			7.16 (3.89)			1.61 (0.58)		
A little	22.67 (12.56)			8.26 (4.32)			1.95 (0.66)		
Moderate	28.33 (14.64)			10.38 (5.05)			2.37 (0.78)		
A lot	32.46 (17.20)			12.47 (5.86)			2.78 (0.86)		
Education		<0.0001	0.02		<0.0001	0.06		<0.0001	0.05
High school or less	23.89 (12.26)			8.95 (4.56)			2.08 (0.76)		
Apprenticeship, technical institute, trade or vocational school, college, CEGEP or other non-university certificate or diploma	26.80 (15.68)			10.46 (5.26)			2.23 (0.88)		
University certificate, diploma or degree	21.78 (13.02)			7.69 (4.47)			1.84 (0.70)		
Immigrant status		<0.0001	0.02		<0.0001	0.01		<0.0001	0.04
First generation	21.03 (12.48)			8.44 (4.30)			1.80 (0.66)		
Second generation	23.21 (12.13)			8.16 (4.48)			1.91 (0.72)		
Third generation or more	25.36 (15.11)			9.27 (5.28)			2.15 (0.85)		

**Table 3:** Multivariate regressions of support for violent radicalization (SyfoR) on endorsement of COVID-19 conspiracy theories and psychological distress, with and without a conspiracy theory * psychological distress interaction term.

	β	95% CI		*p*-Value	β	95% CI		*p*-Value
Intercept	6.68	4.45	8.91	<0.0001	17.6	14.45	20.74	<0.0001
Endorsement of COVID-19 consp. theories	0.88	0.80	0.96	<0.0001	−0.21	−0.42	0.01	0.06
Psychological distress (mean score)	6.32	5.77	6.86	<0.0001	1.10	0.12	2.08	0.03
Self-reported gender (ref = woman)								
Man	4.38	3.74	5.02	<0.0001	3.66	2.98	4.35	<0.0001
Gender-diverse	7.45	3.06	11.84	0.01	7.98	3.63	12.33	0.0004
Age	−0.29	−0.36	−0.22	<0.0001	−0.29	−0.36	−0.22	<0.0001
City (ref = Montreal)								
Calgary	2.18	1.27	3.09	<0.0001	2.55	1.63	3.47	<0.0001
Edmonton	1.76	0.88	2.65	0.0001	2.03	1.17	2.90	<0.0001
Toronto	1.39	0.65	2.14	0.0003	1.70	0.96	2.44	<0.0001
Financial problems (ref = Not at all)								
A little	0.10	−0.66	0.86	0.79	0.42	−0.31	1.15	0.26
Moderate	0.93	−0.05	1.90	0.06	1.21	0.24	2.17	0.01
A lot	0.39	−0.82	1.61	0.52	−0.33	−1.56	0.90	0.59
Education (ref = High school or less)								
Apprenticeship, Tech. school or vocational school, college, CEGEP or other non-university cert. or diploma	1.55	0.71	2.39	0.0003	1.02	0.20	1.84	0.02
University cert., diploma or degree	2.18	1.38	2.98	<0.0001	1.42	0.62	2.22	0.0005
Immigration (ref = 3rd generation or more)								
1st generation	−1.54	−2.33	−0.75	0.0002	−1.23	−2.00	−0.45	0.002
2nd generation	−0.04	−0.75	0.66	0.91	0.13	−0.56	0.82	0.71
Interaction Endorsement of COVID-19 consp. theories * Psychol. distress					0.49	0.40	0.57	<0.0001

Note. CI: confidence interval.

**Table 4:** Moderation of the association between endorsement of COVID-19 conspiracy theories and SyfoR by psychological distress.

Outcome	Moderator (Level)	Estimate	95% CI	*p*-Value
SyfoR	Psychological distress (≤1.75)	0.47	0.35–0.59	<0.0001
	Psychological distress (>1.75)	1.36	1.26–1.46	<0.0001

Note. CI: confidence interval.

## Data Availability

Data is available from the authors upon request.

## Supplementary Materials

The following are available online at https://www.mdpi.com/article/10.3390/ijerph18157846/s1, Table S1: Multivariate regressions of RIS, Table S2: Moderation of the association between endorsement of COVID-19 conspiracy theories and RIS by psychological distress.
