# Supplementary material for: Conspiracy Theories, Psychological Distress, and Sympathy for Violent Radicalization in Young Adults during the COVID-19 Pandemic: A Cross-Sectional Study
Source: Int J Environ Res Public Health. 2021 Jul 24;18(15):7846. doi: 10.3390/ijerph18157846 (PMC8345664; doi:10.3390/ijerph18157846)
Supplement: Supplementary file 1 [file ijerph-18-07846-s001.zip › ijerph-1268613-supplementary.pdf]

Supplemental Table S1. Multivariate regressions of RIS

	β	95% CI		*p*-value	β	95% CI		*p*-value
**Intercept**	7.17	5.86	8.47	<.0001	11.20	9.57	12.84	<.0001
**Endorsement of COVID-19 consp. theories**	0.41	0.37	0.46	<.0001	0.01	-0.09	0.12	0.79
**Psychological distress (mean score)**	2.91	2.58	3.24	<.0001	0.99	0.49	1.49	0.0001
**Self-reported gender (ref = woman)**								
Man	1.69	1.36	2.02	<.0001	1.43	1.09	1.77	<.0001
Gender-diverse	4.62	2.10	7.14	0.0004	4.81	2.31	7.32	0.0002
**Age**	-0.20	-0.24	-0.16	<.0001	-0.20	-0.24	-0.16	<.0001
**City (ref = Montreal)**								
Calgary	0.68	0.16	1.20	0.01	0.81	0.30	1.33	0.002
Edmonton	0.71	0.22	1.21	0.005	0.81	0.32	1.30	0.001
Toronto	0.79	0.36	1.21	0.0003	0.90	0.48	1.32	<.0001
**Financial problems (ref = Not at all)**								
A little	0.49	0.06	0.93	0.03	0.61	0.19	1.03	0.005
Moderate	0.60	0.06	1.14	0.03	0.70	0.16	1.25	0.01
A lot	0.44	-0.25	1.13	0.21	0.17	-0.52	0.87	0.62
**Education (ref = High school or less)**								
Apprenticeship, Tech. school or vocational school, college, CEGEP or other non-university cert. or diploma	0.81	0.33	1.29	0.001	0.61	0.14	1.09	0.01
University cert., diploma or degree	1.21	0.74	1.68	<.0001	0.93	0.46	1.41	0.0001
**Immigration (ref = 3rd generation or more)**								
1st generation	-0.29	-0.71	0.13	0.18	-0.17	-0.60	0.25	0.42
2nd generation	0.26	-0.16	0.68	0.22	0.32	-0.09	0.74	0.13
**Endorsement of COVID-19 consp. theories * Psychol. distress**					0.18	0.14	0.22	<.0001

Note. CI: confidence interval; RIS: Radicalism Intention Scale

Supplemental Table S2. Moderation of the association between endorsement of COVID-19 conspiracy theories and RIS by psychological distress

Outcome	Moderator (level)	Estimate	95% CI	*p*-value interaction
**RIS**	Psychological distress (≤1.75)	0.27	0.20 – 0.35	<.0001
	Psychological distress (>1.75)	0.62	0.57 – 0.67	<.0001

Note. CI: confidence interval; RIS: Radicalism Intention Scale
